# Self-insight in probabilistic categorization – not implicit in children either

**DOI:** 10.3389/fpsyg.2014.00233

**Published:** 2014-03-20

**Authors:** Ferenc Kemény

**Affiliations:** ^1^Department of Cognitive Science, Budapest University of Technology and EconomicsBudapest, Hungary; ^2^Research Institute for Linguistics, Hungarian Academy of SciencesBudapest, Hungary

**Keywords:** implicit learning, explicit learning, probabilistic categorization, weather prediction task, children, development, self-insight

## Abstract

The weather prediction (WP) task is a probabilistic category learning task that was designed to be implicit/procedural. In line with this claim, early results showed that patients with amnesia perform comparably to healthy participants. On the other hand, later research on healthy adult participants drew attention to the fact that the WP task is not necessarily implicit. There have been results showing that participants can access structural information acquired during the task. Participants also report that their responses are based on memories and rule knowledge. The contradictory results may be reconciled by assuming that while explicit learning occurs on the WP task in case of adults, in children the learning process is implicit. The present study aims at testing this hypothesis. Primary school children completed the WP task; the experimental group performed the original task, whereas in the control group cues and outcomes were associated on a random basis, hence their version of the WP task lacked a predictive structure. After each item, participants were asked whether they relied on guessing, intuition, “I think I know the answer” type of knowledge, memories of previous items, or knowledge of rules. Self-insight reports of the experimental group were compared to a control group. Results showed that children learn similarly to adults: they mostly (but not completely) rely on explicit, and not on implicit processes.

## SELF-INSIGHT IN PROBABILISTIC CATEGORIZATION – NOT IMPLICIT IN CHILDREN EITHER

Traditionally cognitive psychology interprets human memory as a fractionated system. A crucial differentiation is related to our ability to consciously access stored representations; representations that may be accessed consciously are considered explicit, while those that are unavailable for conscious access are implicit. Previous studies employed a number of different methods to assess implicit learning, which is defined as the incidental acquisition of complex information with the inability of verbal recall (Reber, 1993). Some of these methods – e.g., the serial reaction-time task (Nissen and Bullemer, 1987) or artificial grammar learning (Reber, 1967) – focus on the acquisition of sequential regularities, while others test categorization. The current study uses the latter approach, and employs the weather prediction (WP) task (Knowlton et al., 1996). In the WP task, participants receive one, two or three out of four different cues. Their goal is to guess whether there would be rain or sunshine. Unknown to the participants, each cue is associated with the outcomes with a given probability. During the task, participants’ performance improves from chance to 70–80% correct.

Early results on the WP task were obtained from neuropsychological patients. One of the greatest appeals of implicit learning paradigms is that they show that patients with amnesia are able to acquire environmental information despite their severe declarative deficit. Early studies using the WP task demonstrated that early in the task the performance of patients with amnesia is comparable to that of healthy participants, but in the later phases their learning effect disappears. At the same time, they show a floor effect on the debriefing questions (including questions about the number of different cues, Knowlton et al., 1994). Amnesic patients are often contrasted with patients of Parkinson’s disease (PD), who can be characterized with an impaired implicit memory system. In accordance, PD patients show the opposite pattern as the Amnesia group: their performance on the debriefing task is identical to that of healthy participants, while their categorization is not better than chance, especially in the early phases of the task (Knowlton et al., 1996). These results suggest that early in the task participants rely on their procedural systems, while the later phases tap declarative memory. This hypothesis is identified as the “implicit first hypothesis” (Kemény and Lukács, 2013).

Subsequent neuropsychological studies focused on qualitative analysis of the learning process and hypothesized a different mapping between performance and memory systems. A study by Gluck et al. (2002) identified three different types of strategies, the “one-cue strategy,” the “singleton strategy,” and the “multi-cue” strategy. In the case of the one-cue strategy, participants focus on a specific cue and give a consistent answer in the presence of that cue, but respond randomly when it is absent. In the case of the singleton strategy, participants respond consistently if there is only one-cue in the item, but respond randomly in the case of combinations. The multi-cue strategy is the optimal strategy. Participants using the multi-cue strategy focus on all appearing cues, they analyze each cue, combine the mean predictive value and make a decision based on the average. The two suboptimal strategies are similar, since in both cases participants need to focus on one-cue at a time (single strategies), while in the optimal strategy they focus on all cues present. According to Gluck et al. (2002) this difference in complexity leads to differences in explicitness: strategies using one-cue at a time are easy to verbalize, hence they can be considered explicit, while the multi-cue strategy is difficult to verbalize, hence it is expected to be implicit. Note that strategy use by itself is surmised to be implicit: Gluck et al. (2002) demonstrated a lack of correlation between real strategy use and self-insight on strategy use. Healthy participants are expected to develop an initial single strategy, and switch to the optimal multi-cue strategy later on. There is neuropsychological evidence supporting this “strategy hypothesis”: results show that patients with hypoxia have difficulties in developing the basic (explicit) strategies, while PD patients are capable of developing explicit strategies, but are unable to switch to more advanced cue-utilization.

A different line of research tested the relationship between performance and self-insight. If performance is in line with self-insight, the processes involved are considered explicit, while incongruence reflects implicit functioning. Previous studies of the WP task found that while participants show consistent strategy use, they are mostly unable to report it, and even if they do report strategy use, it is very unlikely they report the one they had actually employed (Gluck et al., 2002). However, this result does not necessarily reflect implicit learning as self-reports only reveal strategy use, no structural information is collected about acquired representation (in the current case: about the strength of association between cues and outcomes). Subsequent experimental studies, however, did test structural knowledge (Lagnado et al., 2006; Newell et al., 2007). In two experiments Lagnado et al. (2006) tested blockwise (Experiment 1) and itemwise (Experiment 2) whether participants are able to give an account of the predictive value of each cue, and how important they find the cues in prediction. Results showed that participants learned the strong predictive cues very early, and soon realized the importance of these cues. Weak cues were learned later and were considered less important. These results show that healthy participants rely on explicit mechanisms during the WP task.

Experiment 2 by Newell et al. (2007) provided similar results: in this study participants faced one of two versions of the WP task and had to provide the same probability and cue-usage ratings as in the Lagnado et al. (2006) study. The two versions of the WP task were feedback-based and observational. The former was identical to the one used in previous studies, while in the latter participants were not required to guess the weather as cues and outcomes were shown simultaneously. The former is considered a procedural, while the latter a declarative version of the WP task. Results were similar in both conditions and were also in concert with the previous study: participants learned the stronger cues first and reported that these stronger cues were more important. These studies support the so-called explicit hypothesis (see Kemény and Lukács, 2013), since they suggest that learning on the WP task is completely explicit.

The Lagnado et al. (2006) and Newell et al. (2007) studies focus on structural information. That is, these studies collect information from participants about their knowledge. An interesting phenomenon that previous studies have shown is that – despite their severe difficulties with declarative learning – patients with amnesia are able to develop some kind of structural knowledge: they can provide information on how cues and outcomes are related to each other (Reber et al., 1996).

A previous study by Dienes and Scott (2005) looked at whether participants were conscious that their judgment constituted knowledge, and also conscious of the structural regularities that enabled those judgments. Dienes and Scott tested participants with an artificial grammar learning task. Participants had to report whether they relied on guessing, intuition, pre-existing knowledge, task-based memories or task-based rule-knowledge during each grammaticality judgment. In a previous study we adapted this method to the WP task (Kemény and Lukács, 2013); there was no sign of the involvement of implicit processes in learning on the WP task. Participants did not perform better than chance with “implicit” type of answers (“guessing,” “intuition,” “I think I know the answer”), while on items associated with “explicit”-type of answers (“I remember,” “I know the rule”) their performance was high. In sum, studies testing conscious access tend to show that learning on the WP task is explicit.

### THE CURRENT STUDY

In spite of the extensive literature on different aspects of learning on the WP task, relatively little is known about children’s strategies. In the current study we tested children on the WP task and also collected self-insight reports after each decision to find out whether the task is suitable for measuring implicit learning in children. As explained above, there are three hypotheses concerning learning on the WP task. The implicit first hypothesis (Knowlton et al., 1994) suggests that learning is implicit in the early phases of the task and it only becomes explicit in the later phases. Following this assumption, one expects the number of explicit self-insight reports to continuously increase with time. However, one should also expect above chance performance on early items with implicit responses.

The second option is the strategy hypothesis (Gluck et al., 2002). This theory expects participants to differ in their explicit awareness of the strategy they use. Users of the single strategies are expected to show explicit awareness, while users of the multi-cue strategy should solve the task in an implicit manner. Based on this account one expects more implicit answers for the multi-cue strategy. Another possibility is that the number of implicit answers is comparable for both strategies, but performance is higher for implicit items in multi-cue users and for explicit items in single strategy users.

The third account is the explicit hypothesis (Lagnado et al., 2006; Newell et al., 2007) which suggests that learning is explicit throughout the WP task. This account predicts the number of explicit answers to be consistent throughout the task, but performance is expected to improve with time. At the same time, performance on guessing and intuition items (implicit class) are not expected to be better than chance. Another possibility is that the number of explicit items increases with explicit-associated performance constantly above, and implicit-associated performance constantly at chance level.

## MATERIALS AND METHODS

### PARTICIPANTS

Fifty-seven children participated in the study. All children were pupils of one of two primary schools, their mean age was 9.79 (SD = 1.36, min = 6.92, max = 12.58). Children were randomly assigned to one of the two conditions (28 in the experimental and 29 in the control condition). Prior to the study, parents of participants provided a written informed consent in accordance with the principles set out in the Declaration of Helsinki and the stipulations of the local institutional review board. All participants were tested individually in a quiet room in their school.

### DESIGN AND STIMULI

From the participants’ perspective the two conditions were identical. Participants had to solve a WP task (identical to the one used in Kemény and Lukács, 2010; based on Knowlton et al., 1994) in which one, two or three out of four different cues were shown, and they had to decide whether the cue combination would lead to sunshine or rain. Below the cues there were two images: one showing sunshine, the other showing rain. Participants had to click on the image representing the assumed outcome. Immediately after their response feedback was provided: the incorrect answer disappeared and only the correct outcome remained on the screen along with the cues, and it also disappeared after 1500 ms.

We collected subjective self-insight measures after each item (through a procedure based on Dienes and Scott, 2005; identical to the one used in Kemény and Lukács, 2013). Participants faced the following question: “How sure were you in your decision?” Using the mouse, participants had to answer by clicking on a straight line. Five statements were indicated above the line: “I was guessing,” “It was intuition,” “I think I know the answer,” “I remember the answer,” “I know the rule.”^1^ As soon as the participant provided a response a new item appeared.

The design of the WP task is identical to the one used in previous studies by Kemény and Lukács (2010, 2013). There were two conditions: an experimental and a control condition. In the experimental condition there were two strong and two weak cues for each outcome. Cue1 was a square predicting sunshine in 85.7% of the time (note that all other appearances led to rain); Cue2 was a triangle associated with sunshine in 70% of cases; Cue3 was a pentagon predicting rain in 70% of the time; and Cue4 was a rhombus associated with rain in 85.7% of cases. Children in the control condition faced a very similar task: the cues and cue-combinations were presented in the same order as in the experimental condition, but each cue and cue-combination was randomly associated with the outcomes. **Table 1** provides the predictive value of each cue and cue-combination in the two tasks. Participants faced four blocks of 50 trials, trials were in the same pseudorandom order for all participants with the restriction that no combination could appear in two consecutive trials.

**Table 1:** Types and occurrences of cues or cue-combinations per blocks of 50 trials in the two conditions.

Cues	Frequency	Experimental condition	Control condition
A	8	0.875	0.5
B	4	0.75	0.5
C	4	0.25	0.5
D	8	0.125	0.5
AB	8	0.875	0.5
AC	1	1	1
BC	2	0.5	0.5
BD	1	0	0
CD	8	0.125	0.5
ABC	2	1	0.5
ABD	1	1	1
ACD	1	0	0
BCD	2	0	0.5

*
The first column (Cues) shows which cues are present in a given combination: A is cue1, B is cue2, C is cue3, D is cue4. Frequency is the number of appearances within a block of 50 trials. The third and fourth columns provide the probability that the given cue or combination leads to sunshine in the two conditions.*

## RESULTS

### DATA ANALYSIS

Accuracy in the WP task is the ratio in which participants make the correct prediction based on the combined probability. That is, if cues 1 and 3 are present, the average predictive value of sunshine is (85.7 + 70)/2 = 77.85%, which is above chance level, hence we take sunshine as a correct response. Correct responses are based on predictive values and not on feedback. Hence it is possible that an answer is correct even if it is not the same as the outcome.

In self-insight reports, there are five different response categories. In order to analyze performance associated with the five categories (guess, intuition, think, remember, rule) by the early (Block 1) and late (Block 4) phases valid data in all 10 cells is required. Previous studies (Dienes and Scott, 2005; Kemény and Lukács, 2013) reported that this pattern of data was only available for a small number of participants. For this reason, categories are combined into classes: one in which participants had explicit representations on the basis of their knowledge (explicit category), and one in which they did not have any (implicit category). The latter involves the guess and intuition, while the former involves the remember and rule reports. think-type answers are analyzed as a separate, transitional class. This analysis requires valid data in all six cells. Some participants however did not meet this criterion, and those who had empty cells were excluded from all analyses. As a result, all analyses presented below include data from fifteen participants in the experimental condition and seventeen participants in the control condition.

Previous studies highlighted the importance of different strategies in the WP task (Gluck et al., 2002; Hopkins et al., 2004; Shohamy et al., 2004). According to these studies, the implicit vs. explicit nature of learning differs according to the strategy used. In the following section we present analyses comparing the self-insight of participants using different strategies. The identification of the strategies is identical to previous studies (Gluck et al., 2002; Kemény and Lukács, 2013): a model score (see equation 1) is computed for each strategy. multi-cue strategy is granted if the model score for the multi-cue strategy is below 0.1. If the multi-cue model score is above 0.1, but one of the single strategy model scores is below 0.1, we assume that single strategy was used. If no model scores are below 0.1, we do not attribute any strategy. To avoid multiple analysis of the same data, we tested strategy use on results from Block 2 (this analysis is identical to Kemény and Lukács, 2013).

(1) $$ModelScore_{M}=\frac{\sum_{P}{\left(\#sun\_\exp ected_{P,M}-\#sun\_actual_{P}\right)}^{2}}{\sum_{P}{\left(\#presentations_{p}\right)}^{2}}$$

### LEARNING PERFORMANCE

**FIGURE 1:**
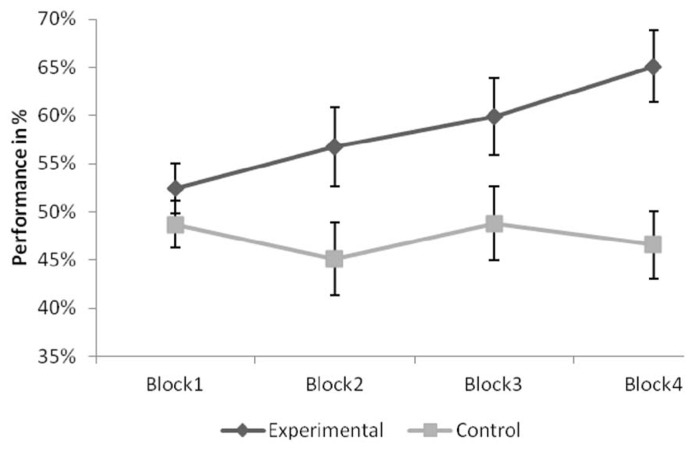
**Hit rates in the two conditions throughout the four blocks in the experimental and the control group.** Error bars indicate Standard Errors.

Learning performance was only analyzed in the experimental group, as there was no probabilistic structure in the control condition. There was a monotonic increase in performance, illustrated by a significant linear polynomial trend, *F*(1,14) = 11.827, *p* < 0.01, $\eta_{p}^{2}$ = 0.458. **Figure 1** shows performance by block for both the experimental and the control group.

### COMPARING EARLY AND LATER BLOCKS IN TERMS OF SELF-INSIGHT BY CONDITION

As explained above, the implicit-first hypothesis suggests that learning on the WP task is implicit in the beginning and becomes explicit later on (Knowlton et al., 1994, 1996). The strategy hypothesis predicts the reverse pattern (Gluck et al., 2002). Similarly, to our previous study (Kemény and Lukács, 2013) we identify Block 1 as the “early phase,” and Block 4 as the “late phase.” First we compare the number of different answers given by the two groups. A 2 × 3 × 2 repeated measures ANOVA was conducted with Block (early vs. late) and answer-type (implicit vs. think vs. explicit) as within-subject variables and condition (experimental vs. control) as between subject variable. As the analysis is conducted on the number of cases, the number of cases in each Block (48) and each condition (192) is invariant (note that there were two items with a predictive value of 0.5 in each block; as there is no correct prediction for these items, they were excluded from the analysis).

**FIGURE 2:**
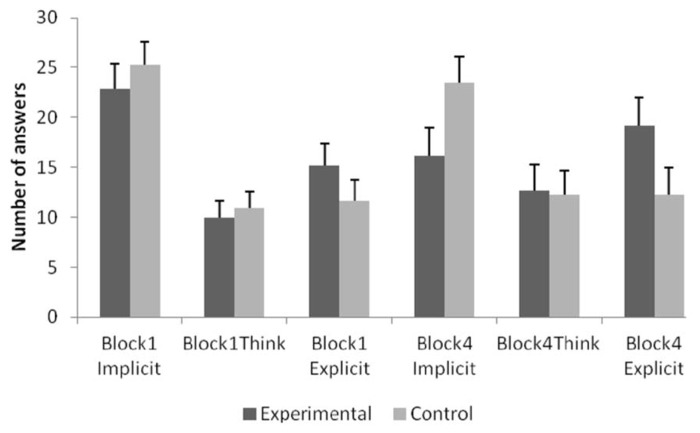
**The number of implicit and explicit type of answers in Blocks 1 and 4 by condition.** Error bars indicate Standard Errors.

Results showed that there was a significant main effect of answer type, *F*(2,60) = 9.224, *p* < 0.001, $\eta_{p}^{2}$ = 0.235. Bonferroni corrected *post hoc* tests revealed that the number of implicit answers are significantly higher than both the number of think-type answers (*p* < 0.00033) and the number of explicit answers (*p* < 0.016). The number of explicit and think-type answers did not differ from each other significantly (*p* = 0.214). No other effect was significant. The Block × Type interaction was approaching significance, but since it does not reflect condition-based changes, it is not discussed any further. **Figure 2** provides the number of implicit vs. think vs. explicit answers by Block and by condition.

### CATEGORIZATION PERFORMANCE ASSOCIATED WITH SELF-INSIGHT TYPE BY CONDITION AND BY BLOCK

In the next step we tested whether participants’ performance differed by answer type. That is, whether the observed performance in the two groups differed based on their association with implicit or explicit self-report. A 2 × 3 × 2 repeated measures ANOVA was conducted with Block (early vs. late) and answer type (implicit vs. think vs. explicit) as within-subject variables and condition as between-subject variable. The dependent variable was performance, which is the mean ratio of correct answers.

Results revealed that performance differed in the two conditions, as shown by a significant main effect of condition, *F*(1,30) = 9.393, *p* < 0.01, $\eta_{p}^{2}$ = 0.238. Data shows that performance of the experimental group was higher than that of the control group. See **Figure 1** for details. Results also showed that this advantage was not stable in time, as revealed by a significant Block × Condition interaction, *F*(1,30) = 6.375, *p* < 0.05, $\eta_{p}^{2}$ = 0.175. At the same time, the main effect of Block was not significant, *p* = 0.114. There was a significant main effect of answer type, *F*(2,60) = 3.521, *p* < 0.05, $\eta_{p}^{2}$ = 0.105. Bonferroni corrected *post hoc* comparisons revealed that performance on the explicit items was significantly higher than performance on the implicit items (*p* < 0.016). No other difference was significant (*p* = 0.45 for implicit and think answers, *p* = 0.868 for think and explicit answers). No other effects were significant (all *p*s > 0.1).

To further analyze the Block × Condition interaction, a separate one-way ANOVA was conducted for each Block with condition as between-subject variable. The ANOVA revealed no significant difference between the groups on Block 1, *p* = 0.304, while the experimental group performed significantly higher on Block 4, *F*(1,30) = 13.129, *p* < 0.001, $\eta_{p}^{2}$ = 0.304.

**FIGURE 3:**
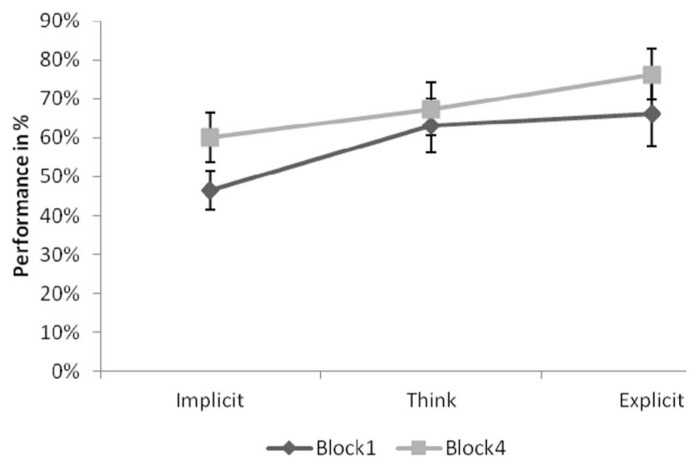
**Performance of the experimental group associated by answer-type in Block 1 and Block 4.** Error bars indicate Standard Error.

To test the pattern of learning in the experimental group, performance associated with each answer-type in each Block was analyzed using a one-sample *t*-test with a target value of 50%. Results showed that performance on implicit-associated items did not differ from chance in both Block 1, *t*(14) = -0.716, *p* = 0.486, and Block 4, *t*(14) = 1.572, *p* = 0.138. In Block 1, performance only marginally differed from chance for both think and explicit-associated items, *t*(14) = 1.907, *p* = 0.077 and *t*(14) = 1.975, *p* = 0.068, respectively. In Block 4 both think and explicit-associated performance was significantly above chance, *t*(14) = 2.535, *p* < 0.05 and *t*(14) = 4.058, *p* < 0.01 respectively. **Figure 3** provides performance associated with each answer-type in both Blocks.

### SELF-INSIGHT BY STRATEGY USE IN BLOCK 2

Whereas in the previous analyses we compared data blockwise (and thus on the time elapse from the onset of the task), in the next session we analyze differences in self-insight by different strategy-use. Gluck et al. (2002) suggests that single strategies are easy to verbalize, hence they are explicit, while the multi-cue strategy is implicit. To avoid multiple analysis of the same dataset, we computed strategy use on Block 2. In the following analyses, only those participants of the experimental condition were considered, who used either of the single strategies or the multi-cue strategy. Out of the 15 participants in the experimental group who were analyzed earlier, five participants developed a single strategy, and four developed the multi-cue strategy.

A 3 × 2 repeated measures ANOVA was used with answer type (implicit vs. think vs. explicit) as within-subject variable and strategy-use (single vs. multi-cue) as between-subject variable. Similarly, to the above analysis, values by strategy-use were invariant. The ANOVA revealed no significant differences (all *p*s > 0.654).

### CATEGORIZATION PERFORMANCE ASSOCIATED WITH SELF-INSIGHT TYPE BY STRATEGY USE IN BLOCK 2

**FIGURE 4:**
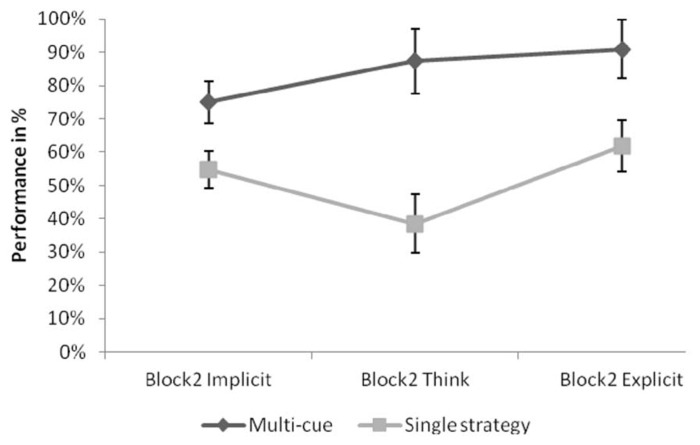
**Performance associated with implicit or explicit self-insight in Block 2 for multi-cue vs. single-cue strategists.** Error bars indicate Standard Errors.

Next we tested performance associated with implicit vs. think vs. explicit answers by strategy use. A 3 × 2 repeated measures ANOVA was conducted with answer type (implicit vs. think vs. explicit) as within-subject variable, and strategy use (single vs. multi-cue strategy) as between-subject variable. The ANOVA revealed a significant main effect of strategy, *F*(1,7) = 115.432, *p* < 0.001, $\eta_{p}^{2}$ = 0.943, with the multi-cue users performing significantly higher than single strategy users. No other effects were significant (both *p*s > 0.327). Data on implicit vs. think vs. explicit-associated performance by strategy use is provided in **Figure 4**.

**Figure 4** shows that in multi-cue users all three answer types were associated with above chance performance. To statistically confirm this observation, we conducted separate one-sample *t*-tests on performance related to each of the three answer types. The target value was 50%. Results showed that all three performance measures differed from chance level: for implicit-associated items *t*(3) = 4.917, *p* < 0.05, for think-answers *t*(3) = 4.823, *p* < 0.05, and for explicit-associated items *t*(3) = 27.13, *p* < 0.001.

## DISCUSSION

The current study was designed to test whether implicit processes underlie learning on the WP task. Results are equivocal: there was no support for the implicit first hypothesis (Knowlton et al., 1996). No significant Block by answer type by condition interaction was observed in either the analysis of the number of responses or the analysis of self-insight related performance.

There is no support for the strategy hypothesis (Gluck et al., 2002) either, as there was no group difference in the number of implicit vs. think vs. explicit self-reports. Results on performance measures show that single strategy users do in fact rely on explicit mechanisms. multi-cue users, on the other hand, utilize explicit strategies much more effectively: their explicit-related performance is higher than that of the single strategy users. The strategy hypothesis predicts high implicit and low explicit performance for the multi-cue users. The results do not confirm this prediction, as performance is slightly higher for the explicit items: there is a general advantage of multi-cue users for both implicit and explicit knowledge. An interesting finding, however, is that multi-cue users show above chance performance for implicit-associated items. This was not found in adults, and it will be discussed in detail below.

On the other hand, results are in line with the predictions of the explicit hypothesis (Lagnado et al., 2006; Newell et al., 2007) since the experimental group shows random performance for implicit-associated items, but a well above chance performance for the explicit-associated ones. This is contrasted with the control group, where participants did not perform better than chance in either type of items. This interpretation is in concert with previous adult data (Kemény and Lukács, 2013), and suggests that children rely on explicit processes while solving the WP task.

As explained in the introduction, the central aim of the current study was to investigate whether the WP task is a suitable task for testing implicit learning in children. The results do not support this hypothesis. Similarly, to adult data, children generally report that they rely on explicit processes when they solve the task. On the other hand – contrary to adults – they also demonstrate an effect of implicit learning, albeit minimal. Our data is in line with previous studies from structural knowledge. As described above, Lagnado et al. (2006) and Newell et al. (2007) argued that participants rely on explicit processes on two grounds: (1) the majority of the participants used a multi-cue strategy very early in the task already, and (2) participants report a reliance on the stronger cues in the early phases, and only later do they turn to use weaker cues. While both results support the involvement of explicit processes, they do not seem to be in line with each other: one cannot use a multi-cue strategy by relying on only two cues that are combined infrequently. The limitation of the Lagnado et al. (2006) study is that they did not measure self-insight. That is what motivated Kemény and Lukács (2013) to adapt the self-insight methodology of Dienes and Scott (2005). Results provided further evidence for the assumption of Lagnado et al. (2006) that (1) there is no qualitative difference between multi-cue and singleton strategies, and (2) the quantitative difference between the strategies shows that the multi-cue strategy is simply more effective than the singleton strategies, and relies more strongly on explicit processes.

### IMPLICIT PROCESSES IN MULTI-CUE USERS

While results do not support the strategy hypothesis, they draw attention to an interesting phenomenon: multi-cue using children perform above chance for implicit-associated items. That is, while our previous study with adults (Kemény and Lukács, 2013) showed no signs of implicit learning at all, and the current study also showed that results in general are in line with the explicit hypothesis, children’s data suggest the involvement of implicit process too. This is especially important in the light of the fact that implicit answers provide one third of responses (18.35 on average, in contrast with a mean of 14.05 think and 18.35 explicit answers). This pattern was not observed in our previous study of adults: in two experiments multi-cue using adults showed approximately the same number of implicit and explicit decisions, with near-chance performance on implicit-associated items (51.99 and 50.45% in Experiment 1 and Experiment 2, respectively^2^).

These results raise the following questions: how can we explain that the first two analyses show no implicit learning, while the second two analyses provide evidence for a strong implicit component? How can we account for the presence of implicit learning in children, but not in adults?

Analyses 1 and 2 contrasted all participants of the experimental and control groups. That is, an experimental group of seventeen and a control group of 22 children were compared. Analyses 3 and 4 on the other hand contrasted users of different strategies in the experimental group. The number of children using different strategies is very low: four children were able to develop multi-cue strategy, while six participants used one of the single strategies. That is, the above chance implicit performance of these four multi-cue users “melted” into the near-chance performance of the remaining 13 participants^2^.

The interesting question concerns age-related changes in the implicitness of learning. There are signs of implicit learning in children, but not in adults. Considering the competitive dual systems approach (Poldrack et al., 1999, 2001) one could argue that explicit learning in adults is more advanced than in children, hence adults do not require implicit mechanisms for solving the WP task, but children do. This would be in line with previous neuropsychological studies showing that when explicit mechanisms are not available (in Amnesia), patients rely on implicit processes. However, since we only tested children, and only a small number of children used a multi-cue strategy, further research is required to answer this question.

### THINK-TYPE ANSWERS

While the classification of guessing, intuition, memory and rule-usage is obvious, think answers were included to make the transition between the implicit and explicit classes smoother. In our previous study (Kemény and Lukács, 2013) this type was a member of the implicit class, as it changed similarly to other types in the same class: it showed a strong decrease from Block 1 to Block 2, and a continuous decay even afterward, while members of the explicit class showed a reverse effect (unpublished pretesting data from Kemény and Lukács, 2013). The current study shows that think-answers behave somewhat differently from the implicit class. **Figure 2** illustrates categorization performance by answer type, and shows that for the experimental group, think-type answers truly deviate from the other classes. Pairwise comparisons show that performance associated with think answers is marginally higher than performance related to implicit answers (*p* = 0.084), but not different from explicit-associated performance (*p* = 0.750). On the other hand, control participants seem to have a lower performance on think answers, but no pairwise comparisons were significant (both *p*s > 0.524).

The analysis of performance related to strategy use revealed a similar pattern. For multi-cue users, think-answers serve as a transition (see **Figure 4**). *Post hoc* pairwise comparison showed that performance associated with this answer type is not different from performance associated with the other classes (both *p*s > 0.346). On the other hand, singleton users seem to have treated think-answers as responses for unexpected errors: results show that think-related performance is marginally significantly smaller than implicit-related performance (*p* = 0.055), whereas it does not differ from explicit-associated performance.

### METHODOLOGICAL CONCERNS

While the current design is adapted from previous studies using the same self-insight methodology (Dienes and Scott, 2005; Kemény and Lukács, 2013), it is still questionable whether introspection can be considered reliable. In the literature of implicit learning (see above), it is well-established that behavior and self-insight are not necessarily correlated. For this reason the current study (similarly to Kemény and Lukács, 2013) compares responses provided by the experimental group to a control condition, and defines explicitness as a deviation from the baseline, rather than as an absolute measure. This may minimize the distortion caused by data originating from introspection.

Another methodological concern is that self-reports are collected after the feedback. The reason for using such a design is that we wanted to keep cue-outcome units together, as previous studies (e.g., Maddox and Ing, 2005) showed that increasing the time asynchrony between cues and outcomes interfere with implicit categorization. Collecting self-reports after the feedback may modify response behavior: participants may alter their self-insight reports based on the outcome. That is, on incorrect responses they may simply report reliance on guessing while they in fact followed a strategy that resulted in an incorrect prediction. *Vice versa*, after guessing led to a correct prediction, they may report that they actually knew the correct answer. At the very extreme, this hypothesis would predict a very low (near 0%) performance for implicit items, and a ceiling effect for the explicit ones, which is not the case. At the same time, the current study does not control for the occasional appearance of this effect.

## CONCLUSION

Based on previous results suggesting that the WP task is not necessarily implicit in adults, the current study tested whether it may be a useful task for the assessment of unconscious learning in children. While there are traces of implicit processing, data strongly support the explicit hypothesis. Children, similarly to adults, report that they relied on memory or knowledge of the rule instead of guessing and intuition. These results complement previous studies testing structural knowledge, and demonstrate that the WP task is not a better measure of implicit learning in children than in adults.

## Conflict of Interest Statement

The author declares that the research was conducted in the absence of any commercial or financial relationships that could be construed as a potential conflict of interest.

## Footnotes

1 Note that both the question and the statements were in Hungarian.

2 Note that in the previous study (Kemény and Lukács, 2013) think-type answers were included in the implicit class.
